# Evaluation of Norepinephrine Transporter Expression and Metaiodobenzylguanidine Avidity in Neuroblastoma: A Report from the Children's Oncology Group

**DOI:** 10.1155/2012/250834

**Published:** 2012-09-25

**Authors:** Steven G. DuBois, Ethan Geier, Vandana Batra, Sook Wah Yee, John Neuhaus, Mark Segal, Daniel Martinez, Bruce Pawel, Greg Yanik, Arlene Naranjo, Wendy B. London, Susan Kreissman, David Baker, Edward Attiyeh, Michael D. Hogarty, John M. Maris, Kathleen Giacomini, Katherine K. Matthay

**Affiliations:** ^1^Department of Pediatrics, UCSF School of Medicine, 505 Parnassus Avenue, M696, San Francisco, CA 94143-0106, USA; ^2^Department of Bioengineering and Therapeutic Sciences, UCSF Schools of Pharmacy and Medicine, San Francisco, CA 94143-0912, USA; ^3^Department of Pediatrics, Children's Hospital of Philadelphia, University of Pennsylvania, Philadelphia, PA 19104, USA; ^4^Epidemiology and Biostatistics, UCSF School of Medicine, San Francisco, CA 94107, USA; ^5^Department of Pathology, Perelman School of Medicine, Children's Hospital of Philadelphia, University of Pennsylvania, Philadelphia, PA 19104, USA; ^6^Department of Pediatrics, University of Michigan Medical School, Ann Arbor, MI 48109, USA; ^7^Department of Biostatistics, University of Florida, Gainesville, FL 32611, USA; ^8^Children's Oncology Group Statistics and Data Center, Arcadia, CA 91006-3776, USA; ^9^Department of Pediatrics, Children's Hospital Boston and Dana-Farber Harvard Cancer Center, Boston, MA 02215-5450, USA; ^10^Department of Pediatrics, Duke University School of Medicine, Durham, NC 27705, USA; ^11^Department of Hematology/Oncology, Princess Margaret Hospital for Children, Perth, WA 6008, Australia

## Abstract

*Purpose*. ^123^I-metaiodobenzylguanidine (MIBG) is used for the diagnostic evaluation of neuroblastoma. We evaluated the relationship between norepinephrine transporter (NET) expression and clinical MIBG uptake. *Methods*. Quantitative reverse transcription PCR (*N* = 82) and immunohistochemistry (IHC; *N* = 61) were performed for neuroblastoma NET mRNA and protein expression and correlated with MIBG avidity on diagnostic scans. The correlation of NET expression with clinical features was also performed. *Results*. Median *NET* mRNA expression level for the 19 MIBG avid patients was 12.9% (range 1.6–73.7%) versus 5.9% (range 0.6–110.0%) for the 8 nonavid patients (*P* = 0.31). Median percent NET protein expression was 50% (range 0–100%) in MIBG avid patients compared to 10% (range 0–80%) in nonavid patients (*P* = 0.027). *MYCN* amplified tumors had lower NET protein expression compared to nonamplified tumors (10% versus 50%; *P* = 0.0002). *Conclusions*. NET protein expression in neuroblastoma correlates with MIBG avidity. *MYCN* amplified tumors have lower NET protein expression.

## 1. Introduction

Metaiodobenzylguanidine (MIBG) is an agent that is specifically taken up by sympathetic nervous system tissues, including neuroblastoma tumors. ^123^I-MIBG plays an essential role in the diagnostic evaluation of patients with neuroblastoma [1]. In addition, high-dose ^131^I-MIBG therapy is an important part of the treatment of patients with relapsed or refractory neuroblastoma [2].

The norepinephrine transporter (NET; encoded by *SLC6A2 *gene) is thought to be the primary transporter responsible for specific active cellular uptake of MIBG [3]. Several studies have demonstrated that neuroblastoma cell lines that lack NET mRNA expression fail to accumulate MIBG [4–6]. NET mRNA levels appear to correlate in vitro with extent of MIBG uptake [6–8]. Moreover, a range of cells that do not typically accumulate MIBG can be engineered to do so by transfection of the NET gene [9–17]. Additional studies in neuroblastoma and other neuroendocrine tumors have suggested that vesicular monoamine transporters (VMATs) and organic cation transporters (OCTs) may also play a role in mediating uptake of MIBG [18–20].

Approximately 10% of patients with neuroblastoma have tumors that do not accumulate MIBG on the basis of negative diagnostic ^123^I-MIBG scans [21]. The determinants of MIBG-avidity in clinical neuroblastoma tumors are unknown. One small study utilized RT-PCR to evaluate NET gene expression in 6 neuroblastoma tumors from patients with negative baseline MIBG diagnostic scans [4]. None of these tumors had detectable NET mRNA, while 90% of the 48 MIBG-avid tumors had detectable NET transcripts. Other groups have not yet replicated these results. Moreover, the correlation between clinical MIBG uptake with NET protein expression and mRNA expression of other putative MIBG transporters has not been studied. The primary aim of the current study was to determine the association between tumor NET mRNA and protein expression with MIBG avidity in patients with neuroblastoma. Secondary aims included evaluation of the association of *SLC6A2 *gene polymorphisms with MIBG avidity, association of mRNA expression for other membrane transporters with MIBG avidity, as well as correlation of NET protein expression levels with clinical features.

## 2. Materials and Methods

### 2.1. Patients and Treatment

Patients were selected from Children's Oncology Group (COG) protocols A3961 and A3973 for the treatment of intermediate-risk or high-risk neuroblastoma, respectively [22, 23]. For the primary analyses focused on NET expression, all patients with institutional report of MIBG nonavid tumors and available tumor mRNA were included (*n* = 29). An unselected group of patients with institutional report of MIBG avid tumors and available tumor mRNA was also included (*n* = 54) to achieve the desired sample size for the primary analyses.

For evaluation of *NET* genotype, patients from A3961 and A3973 who had been included in a previous large-scale neuroblastoma genome-wide association project were included [24, 25]. Patients were treated according to protocol therapy, as previously described [22, 23]. All laboratories performing RT-PCR, immunohistochemistry, and genotyping were blinded to patient characteristics, including MIBG avidity.

All participants or legal guardians provided consent for use of submitted tissue at the time of initial enrollment onto a COG or legacy group neuroblastoma biology study. The COG Neuroblastoma Biology Committee and the UCSF Committee on Human Research approved the study.

### 2.2. MIBG Scans and Central Review

Patients underwent diagnostic ^123^I- or ^131^I-MIBG whole body scans at the time of study entry onto either clinical trial A3973 or A3961. Scans were obtained according to local institutional practice and coded as MIBG avid or nonavid by the treating investigator. Baseline diagnostic MIBG scans were available for central review for 27 patients with high-risk disease treated on protocol A3973 who also had tumor mRNA available for analysis. The primary clinical endpoint for the current study, MIBG avidity, is based upon the results of central review in these 27 patients. Central review was independent of the current study and preceded determination of tumor transporter expression levels for the current study. Investigator assessment of MIBG avidity was used only for evaluation of correlation of NET gene polymorphisms with MIBG avidity.

### 2.3. Quantification of Tumor Transporter mRNA Expression

Neuroblastoma tumor material from patients participating in the COG neuroblastoma biology study was submitted frozen to the Neuroblastoma Tumor Bank at the COG Biopathology Center (Columbus, OH). For tumors with a minimum of 60% neuroblastoma tumor in the submitted material, RNA was extracted using Invitrogen Life Technologies TRIzol Reagent (Total RNA Isolation Reagent; Grand Island, NY). RNA was stored at −80°C until ready for further testing. mRNA quality was assessed using either RNA integrity number (RIN) or ratio of absorbance at 260 and 280 nm (A260/A280). RIN data were available for 17 samples, with a mean RIN of 6.6. For the remaining 66 samples, the mean A260/A280 ratio was 1.62.

Reverse transcriptase PCR (RT-PCR) was performed with Applied Biosystem's (ABI) High Capacity Reverse Transcription Kit using 2 *μ*g of total RNA as per manufacturer instructions. Quantitative real-time PCR (Q-PCR) reactions were conducted with Taqman Fast Universal Master Mix (ABI) in 384-well reaction plates using 10 ng of cDNA per reaction. Commercial TaqMan probe and primer sets for NET (Assay ID: Hs01567441_m1), OCT-1 (Assay ID: Hs00427552_m1), OCT-2 (Assay ID: Hs00533907_m1), OCT-3 (Assay ID: Hs01009568_m1), VMAT-1 (Assay ID: Hs00915193_m1), VMAT-2 (Assay ID: Hs00161858_m1), MATE-1 (Assay ID: Hs00217320_m1), GAPDH (Assay ID: Hs99999905_m1), and PGK1 (Assay ID: Hs99999906_m1) were purchased from ABI. Reactions were run on an ABI Prism 7900HT, and the thermal cycling conditions were 95°C for 20 seconds followed by 60 cycles of 95°C for 3 seconds and 60°C for 30 seconds. Amplification of GAPDH and PGK1 mRNA were performed as internal controls. Gene expression was normalized as the percent of GAPDH and PGK1 expression. Results using either control were well correlated (*r* ^2^ = 0.91) such that only the results normalized to PGK1 are presented. Negative controls with no added cDNA showed no NET amplification. Expression vector containing NET cDNA was used as a positive control and demonstrated that NET expression levels in the tumor tissue were within the linear range of the standard curve. All samples were tested in triplicate, and the result reported is the mean of three separate experiments.

### 2.4. Immunohistochemistry for NET Protein Expression

Paraffin-embedded tumor material was prepared using standard methods at each treating institution and submitted to the COG Neuroblastoma Tumor Bank. Each of the formalin fixed paraffin embedded glass slides was stained with a commercially available NET antibody (NET17-1; MAb Technologies; Stone Mountain, GA) at a 1 : 1000 dilution. Antigen retrieval was performed in a pressure cooker with 0.01 M-citrate buffer at a pH of 7.6. Slides were blocked using the avidin biotin blocking kit (SP-2001; Vector Laboratories; Burlingame, CA). Slides were incubated with the primary antibody for 1 hour at room temperature. The secondary antibody used was a biotinylated anti-mouse IgG 1 : 200 dilution (Vector). Slides were then incubated per manufacturer protocol with an avidin biotin complex (Vector). Lastly, the slides were incubated with diaminobenzidine using a high-sensitivity substrate chromogen system (Dako; Carpinteria, CA) and counterstained with hematoxylin. Pontine tissue and lymph node tissue served as positive and negative controls, respectively. Staining results were assessed by one pathologist (BP) using a four point scoring system: 0+ = tumor cells display complete lack of staining; 1+ = tumor cells show faint cytoplasmic staining; 2+ = tumor cells show intermediate cytoplasmic staining; 3+ = tumor cells display intense cytoplasmic staining (see Supplemental Figure 1 for representative sections in Suplementary Material available online at doi:10.1155/2012/250834.) The percentage of cells positive for NET by IHC was also determined (Supplemental Figure 1). A NET protein expression composite score was derived by multiplying the percentage of tumor cells expressing NET by the intensity score.

### 2.5. *NET* Genotyping

Germline DNA was isolated and genotyped as previously described [25]. In brief, the Illumina HumanHap550 BeadChip was used to genotype samples at over 550,000 single nucleotide polymorphisms (SNPs). From this larger dataset, all SNPs in the NET gene (*SLC6A2*) locus were chosen for analysis in the current study. While no SNPs were excluded due to low minor allele frequencies, only 4 SNPs had minor allele frequencies <10%.

### 2.6. Statistical Methods

Given the sample size and nonnormal distribution of transporter levels, the Wilcoxon rank sum test was used to compare transporter expression levels between patients with MIBG avid versus MIBG nonavid tumors. The Fisher exact test was used to compare NET protein intensity category between patients with MIBG avid vs. MIBG nonavid tumors. The Wilcoxon rank sum test was also used to compare NET protein levels between groups defined by categorical clinical characteristics other than MIBG avidity. Kaplan-Meier methods were used to estimate event-free survival (EFS) from time of entry on to clinical trial (A3961 or A3973) to disease progression, relapse, death from any cause, or second malignancy. Patients without event were censored at time of last followup. Differences in EFS based on median NET protein expression levels were assessed using the log-rank test. *P* values <0.05 were considered statistically significant.

Genotyping data were coded based on the number of copies of the minor allele present at each position evaluated in the NET gene. Binomial tests for equality of proportions were used to evaluate statistical associations using a dominant genetic model, while logistic regression was used to assess an additive genetic model. To adjust for multiple testing, the *R* library [26] “*q*-value” [27] that converts *P* values into corresponding false discovery rates [28] was used.

## 3. Results

### 3.1. Patient Characteristics

Tumor mRNA was available for 83 patients treated on trials A3961 and A3973. One patient with poor mRNA quality was excluded from further analysis. The characteristics of the remaining 82 patients who form the main analytic cohort are shown in Table 1. Thirty-one patients had intermediate-risk and 51 patients had high-risk neuroblastoma. Eighteen percent of patients had tumor *MYCN* amplification. Sixty-four percent of patients in this selected cohort had MIBG uptake on scan by institutional report.

Of the 82 patients with available tumor mRNA, 27 patients had centrally reviewed MIBG scans. Of the 82 patients with available tumor mRNA, 61 also had paraffin embedded tumor available for NET immunohistochemistry. Of these 61 patients, 23 patients had centrally reviewed MIBG scans.

### 3.2. *NET* mRNA Expression Does Not Correlate with MIBG Avidity

**Figure 1:**
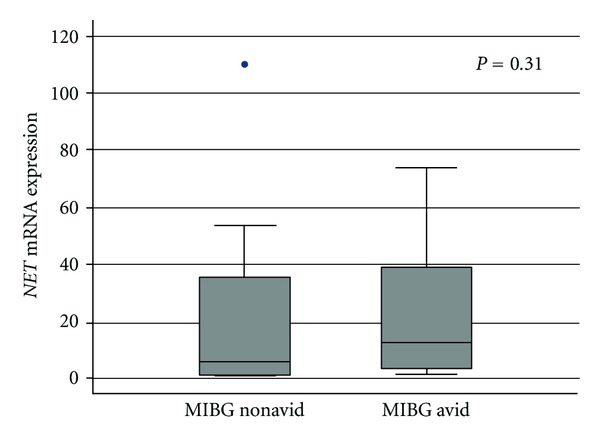
Box plot of tumor *NET* mRNA expression according to tumor avidity for MIBG at initial diagnosis in 27 patients with neuroblastoma (*n* = 19 with MIBG avid tumors and *n* = 8 with MIBG nonavid tumors). End of whiskers represent 5th and 95th percentiles. Transporter expression is expressed as a percent of PGK1 expression.

Based on strong preclinical data implicating NET as the primary transporter responsible for MIBG uptake, we first evaluated the association between *NET *mRNA expression and clinical MIBG avidity. The distribution of *NET* tumor mRNA expression according to centrally-reviewed MIBG avidity is shown in Figure 1. This distribution demonstrates extensive overlap in *NET* expression between patients with and without MIBG avid tumors. The median *NET* expression level for the 19 patients with MIBG avid tumors was 12.9% (range 1.6–73.7%), while the median *NET* expression level for the 8 patients with MIBG nonavid tumors was 5.9% (range 0.6–110.0%; *P* = 0.31). 

### 3.3. Polymorphisms in the *SLC6A2* Gene Do Not Correlate with MIBG Avidity

Germline genotype data for 26 SNPs (Supplemental Table 1) in the *SLC6A2* gene encoding NET were available in 325 patients treated on protocols A3961 and A3973 who were included in a previous genome-wide association study [24, 25]. For this analysis only, investigator assessment of MIBG avidity, rather than central review assessment of MIBG avidity, was used as the clinical outcome of interest. This cohort included 276 MIBG avid and 49 MIBG nonavid patients. There was no correlation between MIBG avidity and any of the SNP genotypes, using either dominant or additive genetic models (Supplemental Table 1).

### 3.4. NET Protein Expression Correlates with MIBG Avidity

**Figure 2:**
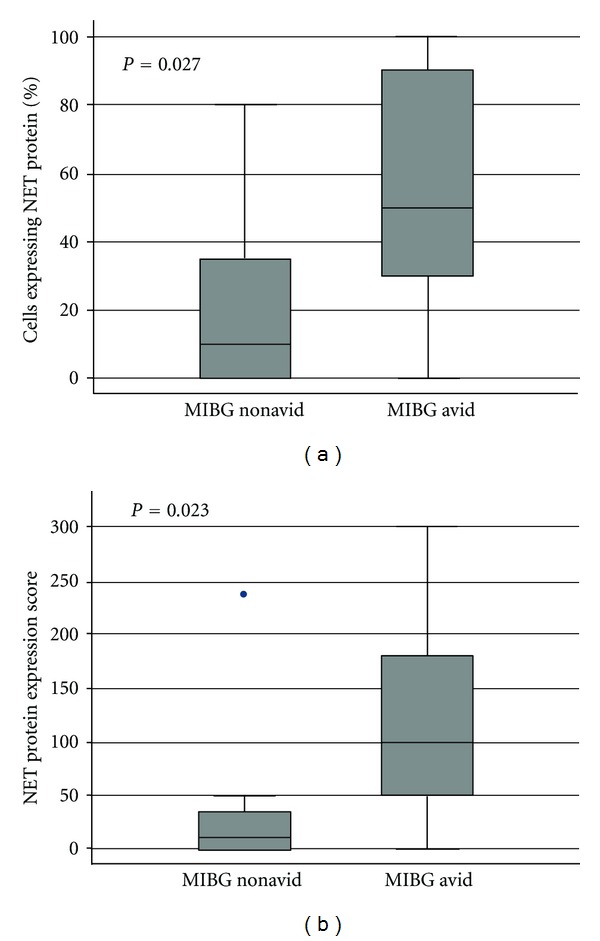
Box plots of percent of tumor cells positive for NET protein (a) and composite NET protein expression score (b) according to tumor avidity for MIBG at initial diagnosis in 23 patients with neuroblastoma (*n* = 15 with MIBG avid tumors and *n* = 8 with MIBG nonavid tumors). End of whiskers represent 5th and 95th percentiles.

Since neither *NET* mRNA expression nor *SLC6A2* genotype correlated with MIBG avidity, we next evaluated whether NET protein expression correlated with clinical MIBG avidity. Of the 82 patients in our overall cohort, adequate archival tumor material was available in 61 cases. Of these 61 patients, 23 had centrally reviewed MIBG scans (15 MIBG avid and 8 MIBG nonavid). The percent of tumor cells with NET protein expression correlated with MIBG avidity. Specifically, the median percent expression was 50% (range 0–100%) in MIBG avid patients compared to 10% (range 0–80%) in MIBG nonavid patients (*P* = 0.027). However, these two groups showed extensive overlap in the distribution of the percent of tumor cells expressing NET, including one MIBG-avid patient with 0% NET expression (Figure 2(a)).

We next evaluated whether intensity of NET protein expression correlates with MIBG avidity. Table 2 shows the distribution of NET immunohistochemistry staining intensity according to MIBG avidity. Patients with MIBG avid tumors had a trend towards higher NET intensity scores compared to patients with MIBG nonavid tumors (*P* = 0.06). Only 1 of 8 patients with centrally reviewed MIBG nonavid tumors had a NET protein intensity score ≥2.

We also evaluated a composite score of percent of tumor cells with NET protein expression and intensity score. This composite score correlated with MIBG avidity. Specifically, the median composite score was 100 (range 0–300) in MIBG avid patients compared to 10 (range 0–240) in MIBG nonavid patients (*P* = 0.023; Figure 2(b)).

### 3.5. *NET* mRNA Expression Does Not Correlate with NET Protein Expression

We next evaluated whether *NET* mRNA expression levels correlated with NET composite protein expression score. A scatterplot revealed little correlation between mRNA and protein expression in 61 patients with both measurements (correlation coefficient = 0.22; *P* = 0.09; Supplemental Figure 2). We also categorized mRNA expression as above and below the group median and evaluated potential differences in NET composite protein score. The median composite protein score for patients with high *NET* mRNA expression was 130 (range 0–300) compared to 90 (range 0–300) for patients with low *NET* mRNA expression (*P* = 0.15).

### 3.6. NET Protein Expression Correlates with Clinical Features in Neuroblastoma

We next evaluated whether NET protein expression composite score correlates with patient clinical characteristics (Table 3). One of the strongest findings was that patients with *MYCN* nonamplified tumors showed significantly higher NET protein expression compared to patients with *MYCN* amplified tumors (*P* < 0.001). Patients with intermediate-risk disease and localized tumors also had significantly higher NET protein expression.

In order to evaluate the prognostic impact of NET protein expression composite score, a Kaplan-Meier curve of estimated EFS was constructed based on transporter expression dichotomized around the group median. Outcomes did not differ by log-rank test (*P* = 0.69; data not shown).

### 3.7. Evaluation of Other Membrane Transporters and MIBG Avidity

While our results demonstrate differential NET protein expression between patients with MIBG avid versus nonavid tumors, we observed substantial overlap in the extent of NET protein expression between these groups. In order to evaluate if other cation transporters might also be responsible for MIBG uptake, we compared mRNA expression for the following transporters between patients with MIBG avid and nonavid tumors: *OCT1; OCT3; MATE1; VMAT1; VMAT2*. While the median *VMAT1 *and *VMAT2* expression levels were higher in MIBG avid tumors, none of these differences were statistically significant (*P* > 0.10 for all comparisons; Table 4).

## 4. Discussion

We report for the first time a positive association between clinical MIBG tumor avidity and NET protein expression by neuroblastoma cells. This finding supports the critical role of NET in mediating specific active uptake of MIBG into neuroblastoma cells [3]. Our results are also consistent with previous preclinical studies demonstrating a correlation between NET protein levels and MIBG uptake into myocardial cells [29].

While we observed a significant association between MIBG avidity and NET protein expression, we also noted overlap in NET protein expression between patients with MIBG avid and MIBG nonavid tumors. Specifically, our cohort includes both patients with high NET protein expression and MIBG nonavid tumors as well as patients with low NET protein expression and MIBG avid tumors. Given that MIBG avidity was centrally assessed by independent review, misclassification of MIBG avidity is unlikely to account for these findings. Our genotyping results suggest that individual variations in the structure of the *SLC6A2 *gene are also unlikely to account for these results.

Instead, our findings raise the possibility that MIBG uptake may be influenced by factors in addition to NET protein. It is possible that tumors with low NET protein expression may accumulate MIBG via other transporters. Candidate transporters include OCTs and VMATs as these transporters have been implicated in MIBG uptake in other neuroendocrine tumors [18–20]. Our evaluation of OCT and VMAT mRNA expression did not reveal any statistically significant associations with MIBG avidity, including subset analysis focusing only on patients with low NET protein expression and MIBG avid tumors (data not shown). Given the trend showing higher *VMAT1 *and *VMAT2* mRNA median levels in MIBG avid tumors, future studies will focus on tumor VMAT protein levels. Likewise, it is possible that tumors with high NET protein expression that fail to accumulate MIBG may have increased expression of MIBG efflux transporters that account for low net uptake of MIBG. While MATE-1 mRNA expression did not correlate with MIBG avidity, evaluation of protein levels of MATE-1 and other efflux transporters may be informative. Alternatively, other physiologic parameters, such as tumor vascularity and pH, may impede distribution of MIBG to the tumor cell membrane. Our results do not address these alternative possibilities and will require further study.

We were unable to replicate previous findings demonstrating an association between NET mRNA expression and clinical MIBG avidity [4]. Several explanations may account for this discrepancy. First, our study relied on archived tumor mRNA obtained within the context of cooperative group trials. As such, degradation of mRNA may have resulted in a false negative result, though our mRNA quality data may argue against this point. Our findings suggest that immunohistochemistry for NET protein may be a more practical approach for evaluating NET expression in future cooperative group studies and in clinical practice. Second, as discussed above, it is possible that the association between MIBG avidity and either NET protein or mRNA expression is imperfect. We note that in previous studies of NET mRNA levels as predictors of clinical MIBG uptake, cases of clinical MIBG avidity in the setting of low NET mRNA expression have been reported [4, 30]. In one previous study, 5 of 11 patients with negative PCR for NET mRNA nevertheless had positive MIBG scans [4]. Third, it is possible that, as we observed, NET mRNA expression does not correlate with NET protein expression in human neuroblastoma tissue, perhaps through posttranslational modification of NET protein.

One of our secondary analyses yielded the previously unreported association between *MYCN *amplification and low NET protein expression. Other statistically significant clinical correlations with lower NET protein expression (patients with high-risk disease and patients with metastatic disease) may be driven by this association with *MYCN* amplification. As an exploratory secondary analysis unadjusted for multiple statistical testing, it is also possible that this association is a chance finding and therefore requires replication by other groups. We note that one previous report did not detect a difference in clinical MIBG avidity according to tumor *MYCN* status [31]. In addition, response rates after high-dose ^131^I-MIBG therapy do not appear to differ between patients with *MYCN *amplified and *MYCN *nonamplified tumors, though all patients were required to have MIBG avid tumors to receive ^131^I-MIBG therapy [32]. The association between *MYCN* status and clinical MIBG avidity will be investigated further in a future analysis by our group. 

## 5. Conclusions

We have demonstrated that neuroblastoma tumor cell NET protein expression correlates with clinical MIBG avidity and also with tumor *MYCN* status. Additional work, including gene and protein expression profiling efforts, will need to investigate other mechanisms of MIBG uptake in cases with low NET protein expression. Future studies may also focus on the correlation between tumor NET protein expression and response to targeted radiotherapy with high-dose ^131^I-MIBG.

## Supplementary Material

The Supplementary Material includes examples of immunohistochemistry staining (Supplemental Figure 1) and the correlation between NET mRNA and NET protein expression (Supplemental Figure 2). The Supplemetary Material also includes the results of genotyping of the gene encoding NET in patients with MIBG avid and non-avid tumors (Supplemental Table 1).

## Figures and Tables

**Table 1:** Characteristics of 82 patients with available tumor mRNA and of the subset of 27 patients with centrally reviewed MIBG diagnostic scans.

	Entire cohort (*N* = 82)	Subset of patients centrally reviewed (*N* = 27)
Median age (range)	11.9 months (0.1–171.7 months)	33 months (3.8–171.7 months)
Age > 18 months at diagnosis	35/82 (42.7%)	22/27 (81%)
Intermediate-risk group : High-risk group	31 : 51	0 : 27
Adrenal primary tumor	38/82 (46.3%)	16/27 (59%)
Stage 4 : Stage 4S : Localized tumors	39 : 7 : 36	15 : 2 : 10
*MYCN*-amplified tumor	15/82 (18.3%)	14/27 (51.8%)
Unfavorable histology	29/80 (36.3%)	24/26 (92.3%)
Elevated baseline urine catecholamines	20/31 (64.5%)^a^	16/27 (59.3%)
MIBG avid tumor at diagnosis	53/82 (64.6%)^b^	19/27 (70.3%)

^a^Available only in patients with high-risk disease.

^b^Institutional report.

**Table 2:** NET protein expression intensity score according to MIBG avidity in 23 patients with neuroblastoma and centrally reviewed MIBG scans.

	NET protein intensity grading			
	0	1+	2+	3+
MIBG Nonavid	3	4	0	1
MIBG Avid	2	3	7	3

**Table 3:** NET protein expression composite score (*n* = 61) according to baseline patient characteristics.

	*N*	Median NET protein expression composite score (range)
Age ≤ 18 months	32	95 (0–300)
Age > 18 months	29	140 (0–300)
Intermediate-risk group	34	160 (0–300)**
High-risk group	27	60 (0–300)
Localized tumor	29	160 (0–300)**
Metastatic tumor	32	55 (0–300)
Adrenal primary tumor	28	100 (0–300)
Nonadrenal primary tumor	33	100 (0–300)
*MYCN* nonamplified	48	160 (0–300)***
*MYCN* amplified	13	10 (0–140)
Favorable histology	36	160 (0–300)
Unfavorable histology	24	70 (0–300)
Normal urine catecholamines^a^	10	65 (0–240)
Elevated urine catecholamines	17	60 (0–300)

^a^Available only for patients with high-risk disease.

**P < 0.05 for difference in expression between clinical groups.

***P < 0.001 for difference in expression between clinical groups.

**Table 4:** mRNA expression for a panel of cation transporters according to tumor MIBG avidity in 27 patients with neuroblastoma and centrally reviewed MIBG scans.^a^

	MIBG Avid (*n* = 19)	MIBG Nonavid (*n* = 8)
Median *OCT1 *mRNA	0.17%	0.11%
(range)	(0.04–0.48%)	(0.03–0.90%)
Median *OCT3 *mRNA	0.60%	1.3%
(range)	(0.04–127%)	(0–6.47%)
Median *MATE1 *mRNA	5.9%	8.2%
(range)	(0.39–57%)	(0.43–102%)
Median *VMAT1 *mRNA	89%	13%
(range)	(0.68–348%)	(0.43–1590%)
Median *VMAT2 *mRNA	175%	27%
(range)	(2.7–2661%)	(0.98–1253%)

^a^Transporter expression is expressed as a percent of PGK1 expression. *OCT2 *mRNA expression was observed in only two tumors.
