# Supplementary material for: Evaluation of Norepinephrine Transporter Expression and Metaiodobenzylguanidine Avidity in Neuroblastoma: A Report from the Children's Oncology Group
Source: Int J Mol Imaging. 2012 Sep 25;2012:250834. doi: 10.1155/2012/250834 (PMC3463166; doi:10.1155/2012/250834)

Supplemental Table 1. Single nucleotide polymorphisms (SNP) in the *SLC6A2* gene (encoding NET protein) and q-values for the correlation between MIBG avidity and each SNP, using additive and dominant genetic models.

Single Nucleotide Polymorphism	Location	Minor Allele Frequency[a]	Additive Model q-value	Dominant Model q-value
rs2242446	5’ near gene	0.26	0.26	1
rs36030	Intron 1	0.15	0.45	1
rs17307096	Intron 1	0.37	0.25	1
rs3785143	Intron 1	0.10	0.32	1
rs1532701	Intron 1	0.45	0.25	1
rs734980	Intron 1	0.07	0.32	1
rs187715	Intron 2	0.03	[b]	1
rs36026	Intron 2	0.10	0.46	1
rs36024	Intron 3	0.47	0.25	1
rs187714	Intron 3	0.40	0.25	1
rs36023	Intron 3	0.28	0.35	1
rs16955591	Intron 3	0.11	0.46	1
rs3785152	Intron 3	0.11	0.46	1
rs40147	Intron 3	0.31	0.46	1
rs1814270	Intron 3	0.32	0.32	1
rs36016	Intron 4	0.45	0.25	1
rs3785155	Intron 4	0.12	0.25	1
rs1345429	Intron 4	0.49	0.26	1

rs11862589	Intron 4	0.48	0.26	1
rs36013	Intron 4	0.09	0.45	1
rs3785157	Intron 7	0.26	0.25	1
rs5568	Intron 7	0.28	0.30	1
rs1566652	Intron 8	0.28	0.25	1
rs5569	Exon 9[c]	0.27	0.25	1
rs36009	Intron 10	0.09	0.46	1
rs42460	3' Untranslated Region	0.11	0.45	1

[a] From NCBI dbSNP database.

[b] Additive model not applicable as no cases with two copies of minor allele.

[c] Synonymous SNP.

Supplemental Figure 1A

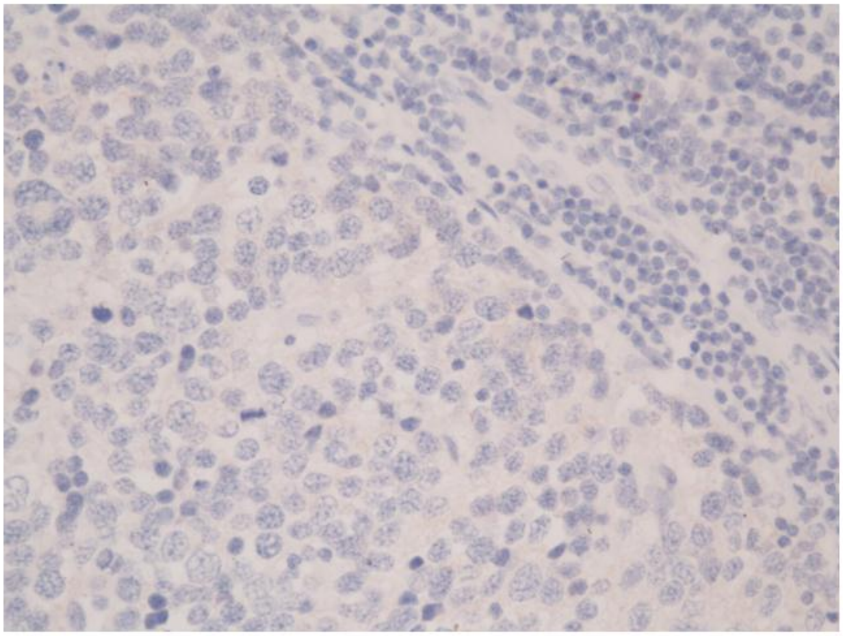

Supplemental Figure 1B

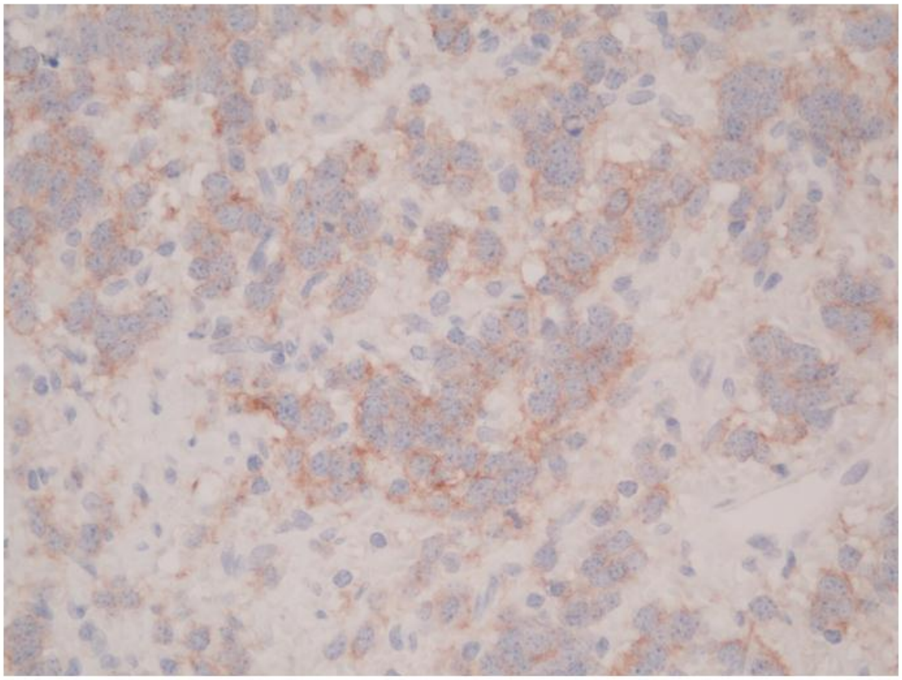

Supplemental Figure 1C

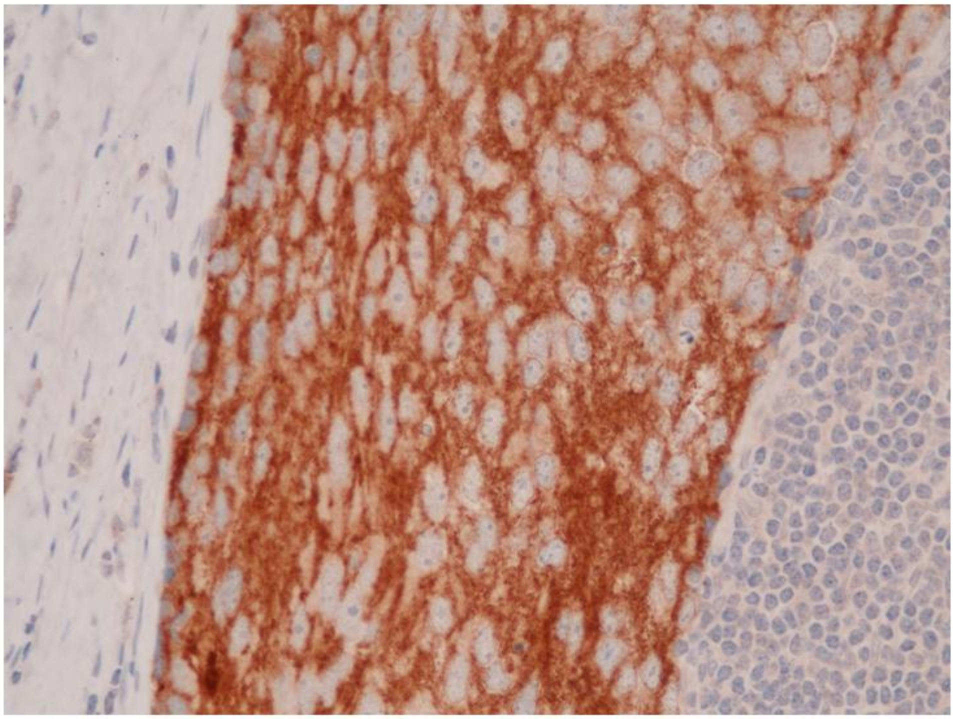

Supplemental Figure 2

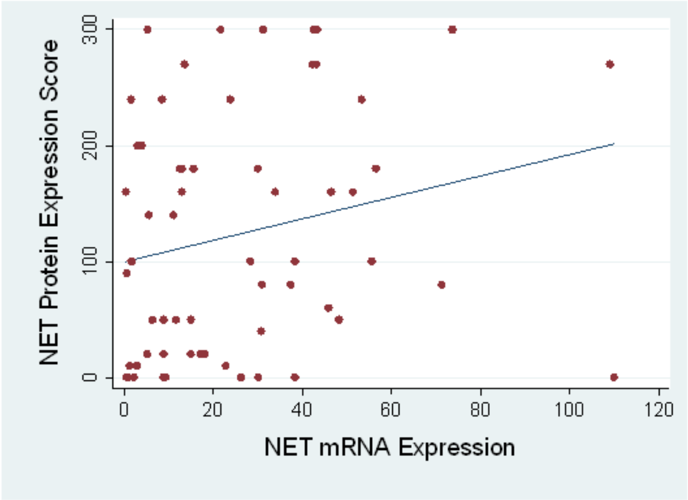

Supplement: Supplementary file 1 — The Supplementary Material includes examples of immunohistochemistry staining (Supplemental Figure 1) and the correlation between NET mRNA and NET protein expression (Supplemental Figure 2). The Supplemetary Material also includes the results of genotyping of the gene encoding NET in patients with MIBG avid and non-avid tumors (Supplemental Table 1). [file 250834.f1.pdf]
